# Leveraging community pharmacies for HIV services in South Africa: Opportunities and constraints

**DOI:** 10.4102/sajhivmed.v26i1.1739

**Published:** 2025-10-28

**Authors:** Angela Tembo, Andy Gray, Tsitsi Nyamuzihwa, Francois W.D. Venter, Jacqueline Maimin, Ahmed Bayat, Jacqui Miot, Deanne Johnston

**Affiliations:** 1 Ezintsha, Faculty of Health Sciences, University of the Witwatersrand, Johannesburg, South Africa; 2 Discipline of Pharmaceutical Sciences, School of Health Sciences, University of KwaZulu-Natal, Durban, South Africa; 3 Independent Community Pharmacy Association, Cape Town, South Africa; 4 Health Economics and Epidemiology Research Office, Faculty of Health Sciences, University of the Witwatersrand, Johannesburg, South Africa

**Keywords:** community pharmacies, HIV services, delivery models, telemedicine, PIMART, legislation, NIMART

## Abstract

Access to HIV services in South Africa remains challenging, despite their availability in the public healthcare sector. While the legislative framework allows for the provision of these services in community pharmacies, the process is often complex.

This article describes various models for the provision of HIV services in community pharmacies in South Africa through a review of existing policies and legislation. It further discusses barriers and opportunities for the expansion of services.

The existing legal framework enables prescribing by healthcare professionals other than medical practitioners through authorisations issued under either the *Medicines and Related Substances Act of 1965* or the *Nursing Act of 2005*.

Community pharmacies have extended their role beyond dispensing medication, with the emergence of telehealth and potential initiatives such as Pharmacist-Initiated Management of Antiretroviral Therapy (PIMART). Telehealth, accelerated by the COVID-19 pandemic, provides remote consultations and electronic prescriptions. PIMART, on the other hand, can empower pharmacists to initiate and manage antiretroviral therapy (ART) for HIV patients, a role traditionally reserved for clinicians. Extending Nurse-Initiated Management of Antiretroviral Therapy (NIMART) into the private sector could further increase ART rollout.

Despite these advancements made in the last two decades, legislative reforms are necessary to fully realise the potential of community pharmacies for providing HIV services.

**What this study adds:** This study highlights the existing legislative framework governing the provision of HIV services in South Africa. It emphasises the critical need for a patient-centred, multidisciplinary approach that leverages the extensive network of community pharmacies nationwide to enhace access and delivery of HIV services.

## Introduction

According to the 2023 Statistics South Africa General Household Survey, 24.6% of respondents first seek healthcare services in the private sector, even though only 15.7% were covered by medical schemes.^1^ Although this implies greater reliance on out-of-pocket payment for health services, it also underscores the importance of private healthcare in South Africa.

Globally, community pharmacies have demonstrated the potential to deliver services promoting health and preventing disease.^2,3^ In many countries, community pharmacists are the most accessible healthcare providers, as they are available without an appointment, are open for extended hours, and are conveniently located.^4^ Examples of pharmacist-initiated services include the provision of emergency post-coital contraception, sexual health products (such as condoms and lubricants), and HIV self-test kits.^5^ In addition, the potential role of community pharmacies in HIV prevention and treatment services is gaining traction.^6,7,8,9^ A single-arm prospective pilot evaluation in Kenya showed that pharmacies have the potential to expand pre-exposure prophylaxis (PrEP) access, which was acceptable to pharmacy providers and clients.^10,11^ A trial in the United States demonstrated that community pharmacy-delivered PrEP was feasible among men who have sex with men.^7^ Locally, a pilot programme conducted through the Southern African HIV Clinician Society (SAHCS), to evaluate an electronic monitoring system, saw over 300 PrEP prescriptions in pharmacies in 2021, using a doctor-supported prescription system.^10^

South Africa has made significant progress toward the Joint United Nations Programme on HIV/AIDS (UNAIDS) 95-95-95 targets, but recent estimates show that there were approximately 178 000 new HIV infections between mid-2023 and mid-2024.^12^ South Africa’s National Strategic Plan for HIV, Tuberculosis, and Sexually Transmitted Infections emphasises broader accessibility and person-centred care.^13^ However, significant barriers to accessing healthcare services persist, particularly in underserved communities and hard-to-reach populations.^14,15,16,17,18^ Harnessing the community pharmacy network, with more than 4000 pharmacies across South Africa, would significantly expand access to antiretrovirals (ARVs).^5,19,20^

This article describes the current legislation applicable to delivering HIV prevention and treatment services in South African community pharmacies, the key roles and competencies required in these roles, and the existing barriers and opportunities in the provision of these services.

## Methods

A desktop review was conducted to synthesise legal, regulatory, and professional guidance relevant to the provision of HIV services through community pharmacies in South Africa. The review technique involved purposive identification and examination of primary legislation, subordinate regulations, and professional guideline documents that define the permissible scope of practice for pharmacists, nurses, and other healthcare providers, as well as standards for telehealth and pharmacy service delivery.

The legislation reviewed included the *Medicines and Related Substances Act* 1965 (Act No. 101 of 1965),^21^ *Nursing Act* 2005 (Act No. 33 of 2005),^22^ the *Pharmacy Act* 1974 (Act No. 53 of 1974),^23^ as well as relevant regulations and scopes of practice. Guideline documents, such as the Rules relating to Good Pharmacy Practice standards, first issued in 2012,^24^ and the Health Professions Council General Ethical Guidelines for Good Practice in Telehealth, issued in 2021,^25^ were also reviewed.

Each document was reviewed in full to extract provisions pertinent to pharmacy-based HIV prevention, testing, and treatment services, with particular attention to task-shifting, prescribing authority, dispensing rights, and service delivery modalities. The analysis aimed to provide an integrated understanding of how the existing legislative and regulatory environment shapes, facilitates, or limits the role of community pharmacies in delivering HIV services, and to identify areas for policy alignment or advocacy to expand access.

### Ethical considerations

This article followed all ethical standards for a research without direct contact with human or animal subjects. Ethical approval for the study under which this analysis is embedded was obtained from the University of the Witwatersrand, Human Research Ethics Committee (HREC) (reference number: 230107) on 28 March 2023.

## Results

South Africa’s National Drug Policy (1996) explicitly states that prescribing rights in primary healthcare should be based on competency rather than professional status.^26^ However, this has not materialised in the South African private health sector, where prescribing is still predominately done by medical practitioners. Section 22A of the *Medicines and Related Substances Act*^21^ allows for authorised prescribers other than medical practitioners and dentists. This provision has enabled the recognition of emergency personnel, optometrists, podiatrists, dental therapists and oral hygienists as authorised prescribers. In addition, section 22A(15) provides for exceptional authorisation to acquire, possess, use, or supply specified medicines. For example, a Primary Care Drug Therapy (PCDT)-trained pharmacist with a section 22A(15) permit is authorised to diagnose, prescribe, and administer medicine for selected conditions, in line with Primary Health Care (PHC) Level Standard Treatment Guidelines (STG) and Essential Medicine List (EML).^26^ This has enabled some private sector community pharmacists to extend their services to improve patient care without compromising patient safety, increase access to healthcare services, increase patient choice, make better use of the skills of health professionals and contribute to the introduction of a more flexible team working in primary healthcare settings.

Nurses have not yet been recognised as authorised prescribers in accordance with the *Medicines and Related Substances Act*.^21,27^ Instead, professional nurses, predominantly in the public sector, have relied on an exception in the *Nursing Act* section 56(6).^22^ Section 56(6) authorisation enables nurses to examine, diagnose, keep prescribed medicines and supply, administer, or prescribe for specified communicable and non-communicable conditions if the services of a medical practitioner or pharmacist are not available. Current regulations limit nurses to medicines up to Schedule 4.^28^ Other nurses, such as those in occupational health services, have relied on section 22A(15) permits.

There is urgent need to expand HIV services, in particular preventative services such as PrEP.^29^ In addition, there is mounting pressure for other healthcare professionals, such as nurses and pharmacists, to initiate and manage patients on antiretroviral therapy (ART) in the private sector.^30,31^

The extensive network of community pharmacies is an untapped resource to augment HIV services.^3^ Delivering HIV services in community pharmacies involves various stakeholders, each playing a critical role in ensuring safe and effective service delivery. The key players in providing services within the community pharmacy ecosystem are the pharmacist, professional nurse, and medical practitioner. As shown in Table 1, significant opportunities in the existing legislative frameworks can be exploited to expand HIV services in the private sector.

**TABLE 1:** Roles of healthcare professionals in HIV testing and counselling as well as initiation and management of antiretroviral therapy.

Healthcare professional and registration	Qualification and additional training required	HIV Testing and Counselling	Prescribe ART	Dispense ART	Permit or licence required
**Pharmacist**					
All pharmacists	Bachelor of Pharmacy	✓	X	✓	-
PCDT	Bachelor of Pharmacy and completion of PCDT-accredited supplementary training	✓	Only PEP for healthcare professionals	✓	Section 22A(15) permit†
PIMART	Bachelor of Pharmacy and completion of accredited PIMART supplementary training	✓	Only 1st-line ARVs for certain patients	✓	Section 22A(15) permit†
**Nurse**					
All professional nurses	Bachelor of Nursing	✓	X	-	-
NIMART	Professional Nurses are required to complete training in NIMART	✓	✓	✓	Section 56(6) authorisation‡
**Medical practitioner**					
All medical practitioners	Bachelor of Medicine and Bachelor of Surgery (MBBCh; MBChB)	✓	✓	Only if in possession of a dispensing licence	Section 22C(1)(a) licence†

ART, antiretroviral therapy; ARVs, antiretrovirals; PEP, post-exposure prophylaxis, PCDT; primary care drug therapy; PIMART, pharmacist-initiated management of antiretroviral therapy; NIMART, nurse initiated management of antiretroviral therapy.

† , South Africa. Medicines and Related Substances Act, 1965 [homepage on the Internet]. Government Gazette No. 101. 1965. Available from: https://www.sahpra.org.za/wp-content/uploads/2020/02/Government_Gazette_Medicines_and_Devices_Act_Jun_2017-1.pdf;

‡ , South African Nursing Council. Nursing Act, 2005 [homepage on the Internet]. Government Gazette No. 33. 2005. Available from: https://www.sanc.co.za/wp-content/uploads/2020/06/Nursing-Act-2005.pdf.

### Pharmacist

In alignment with their scope of practice, a pharmacist is permitted to conduct HIV testing and counselling, dispense prescriptions for ARVs, and engage in health promotion for the prevention and treatment of HIV. As indicated above, pharmacists can render additional services after successful completion of PCDT training and issuance of a section 22A(15) permit by the Director-General (DG).^26,31,32^ However, PCDT pharmacists may not initiate or manage patients on ART, except when providing post-exposure prophylaxis (PEP) to healthcare professionals.^26^

In 2020, the South African Pharmacy Council (SAPC) proposed a scope of practice for pharmacists trained in Pharmacist-Initiated Management of Antiretroviral Therapy (PIMART). Board Notice 101 of 2021 was published for implementation on 13 August 2021.^33^ This notice allows trained pharmacists, with section 22A(15) permits, to perform specific tasks relating to the initiation and management of HIV-positive patients on first-line ART, and the prevention of HIV in patients requesting PrEP and PEP. However, because of legal challenges, PIMART is currently on hold. The Independent Practitioner Association Foundation (IPAF) challenged the suitability of pharmacists to initiate patients on ART through legal action against the SAPC in 2022.^33^ Although the North Gauteng High Court in Pretoria ruled in favour of the SAPC on 14 August 2023, the IPAF appealed this judgement in the Supreme Court of Appeal. The judgement in the appeal case is still awaited.^34^

### Nurse

The *Nursing Act* outlines special provisions relating to authorised nurses working in the public sector and other designated facilities to examine, diagnose, prescribe, and dispense. Nurses who have been authorised in terms of section 56(6) can prescribe and dispense medicines.^22^ In the public sector, the permit is issued by the head of health or delegated medical practitioners at different spheres of government (national, provincial, and local government). In organisations rendering a health service and designated for this purpose by the DG, the medical practitioner in charge issues the permit. Nurses authorised in terms of section 56(6) can prescribe and dispense ARVs in the public sector, provided they have been trained in Nurse Initiated Management of Antiretroviral Therapy (NIMART).

Professional nurses in the private sector may work as independent nursing practitioners or may be employed within health establishments, such as community pharmacies. Those employed in community pharmacies provide various services, including health and wellness services, immunisation, and screening services. Independent nursing practitioners may apply for a section 22A(15) permit from the DG, to acquire, possess, use, or supply specified medicines in Schedules 1–5. However, such permits are mainly limited to services relating to immunisation, family planning, home-based care, occupational health, travel medicine, and haemodialysis. For HIV services, nurses working in community pharmacies may only conduct the initial screening and testing of patients as well as the provision of health education and refer the patient to authorised prescribers, such as medical practitioners, for further care, including ART. Currently, section 22A(15) permits have not been issued to NIMART nurses for the provision of ART in the private sector.

### Medical practitioner

Medical practitioners are permitted to diagnose and prescribe medicine for conditions provided they have adequate training and experience. However, if they wish to dispense, they must complete a dispensing course and obtain a licence in terms of section 22C(1)(a) of the *Medicines and Related Substances Act*.^21^

Additional supplementary training in HIV is available for all healthcare practitioners, such as a diploma in HIV management. However, courses such as these remain optional and do not change the scope of the healthcare practitioners’ practice.

## Models of delivery

Community pharmacies in South Africa have successfully integrated HIV services into their practices, and, over time, HIV-related services have expanded.

### Traditional model of care in community pharmacies

An individual requiring HIV services in the community pharmacy will either self-refer or be referred with a prescription from their medical practitioner for medication. This section will focus on self-referred individuals.

The SAPC allows pharmacies to operate clinics within their facilities, providing ‘accessible, essential clinical services including screening and referral services’.^24^ Pharmacies often employ professional nurses to work in these clinics.

In a community pharmacy, a pharmacist or nurse may conduct HIV counselling and testing (HCT) per their existing scope of practice. Individuals testing positive are referred to a medical practitioner or the public sector for further management. Those testing negative but requiring ARVs for PrEP or PEP will need a prescription from an authorised prescriber. Additionally, HIV self-screening kits are available for sale in community pharmacies. These tests are often purchased off-the-shelf or online without engagement with a healthcare professional.

### Pharmacy-clinic telehealth consultations

The COVID-19 pandemic led to an expansion of digital healthcare services, including consultations with medical practitioners.^25^ In 2021, the Health Professionals Council of South Africa (HPCSA) updated its ethical guidelines on telehealth to include any information and communication platforms used for online consultations between medical practitioners and patients.^25^ However, these ethical guidelines are ambivalent at times. For example, they acknowledge that face-to-face consultations are preferred, and telehealth should be used where there is an existing relationship between the medical practitioner and patient; however, in the event there is not an existing relationship, telehealth can still be provided if it is in the best interests of the patient. The guideline also allows for a wide variety of information and communication technology platforms, excluding social media, if patient consent is obtained and confidentiality is maintained. This has enabled the provision of telehealth consultations from a community pharmacy.

**FIGURE 1:**
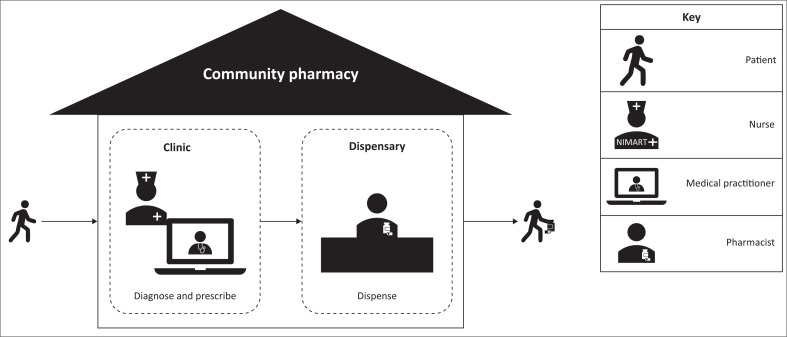
Schematic illustrating the nurse-led pharmacy-clinic telehealth consultations model of care.

Community pharmacies have partnered with individual medical practitioners or telehealth networks to provide pharmacy-clinic telehealth consultations. This model consists of a nurse or pharmacist in a community pharmacy clinic who consults remotely with an online medical practitioner (Figure 1). Telehealth consultations with medical practitioners in a clinic situated in a pharmacy constitute ‘another business or practice in a pharmacy’.^24^ The pharmacy owner must ensure that this business or practice does not affect the quality of the pharmaceutical services. To demonstrate this, amended architectural plans indicating where the other business is situated within the pharmacy are required, as well as payment of the specified application fee to the SAPC. In addition to the costs for the required infrastructure, the pharmacy would partner with a medical practitioner or telehealth network. Establishing and implementing pharmacy-clinic telehealth consultations is thus a costly process.

Although there are no statistics yet on the uptake of such services, they seem to be gaining popularity, with more pharmacies advertising these offerings. The overall cost of the pharmacy-clinic telehealth consultations across the three largest group pharmacies currently varies from ZAR250.00 to ZAR365.00, including the nurses’ and medical practitioners’ fees and exclude any medication or consumables.^35,36,37^

Nurse-led pharmacy-clinic telehealth consultations allow the community pharmacy to be the ‘one-stop shop’ for HIV-related services (Figure 1). Individuals accessing pharmacies for services such as emergency post-coital contraception, family planning services, HIV screening, condoms, as well as prescriptions for sexually transmitted infections, may be referred to the nurse in a consultation room within the pharmacy for HIV screening and testing. In this model, the nurse facilitates a telehealth consultation with a medical practitioner. If ART is required, the medical practitioner sends an electronic prescription to the pharmacy where the medicine is dispensed.

## Discussion

The WHO advocates differentiated service delivery (DSD)^38^ to improve access to HIV medication in resource-limited settings. DSD is a client-centred approach that simplifies and adapts HIV services to the needs of the patients. Both models discussed have successfully adapted HIV care in response to patient requirements. Although telehealth consultations initially emerged as a necessity during the COVID-19 pandemic, their continued integration into healthcare systems suggests they are now an enduring component of service delivery. Additionally, the services provided by the nurse, medical practitioner and pharmacist in both models fall within the scope of practice of each profession.

The South African Department of Health has enabled the collection of repeat prescriptions by stable ART patients at decentralised facilities where they may receive multi-month supplies of medicine.^39^ Unfortunately, medicines for PrEP are not currently part of the Centralised Chronic Medicines Dispensing and Distribution (CCMDD) programme. Private community pharmacies have been included in the pick-up-point options for collecting medication provided through CCMDD. Patients collecting ART from community pharmacies have found it convenient, as queues were far shorter and opening times were extended.^40^ This contracted service is a step in the right direction; however, community pharmacies can offer far more than this. Strategic partnerships with the private sector could improve access, especially services relating to PEP and PrEP.

Unlike the traditional model, pharmacy-clinic telehealth consultations in a community pharmacy call for collaboration between the nurse, medical practitioner and pharmacist to identify persons at risk, conduct the examination and appropriate tests and prescribe and dispense ARV medication in a single facility, normally during the initial visit. Thus, the pharmacy can provide comprehensive primary healthcare services, from consultation to medication dispensing.

Although the pharmacy-clinic telehealth consultations model demonstrates the role community pharmacies could play in delivering HIV-related services, not all pharmacies have the resources to operate them. Furthermore, the associated costs for a telehealth consult could be out of reach for the average South African. The roles of the nurse and pharmacist need to be re-engineered for optimal delivery in resource-constrained settings, such as South Africa.

There is an urgent need for task shifting to permit nurses and pharmacists, through the NIMART and/or PIMART programmes, to prescribe ARVs for the treatment and prevention of HIV in the private sector. This would further facilitate same-day ART initiation.

NIMART has been successful in the public sector; however, the roll-out in the public and private sectors differs considerably, and the current legislation needs to make explicit provision for nurses working in community pharmacies to prescribe ART.^29^

Appropriately trained pharmacists who can initiate and manage patients on ART have been identified as a key resource to help fight HIV.^26,41^ The decision of the courts on whether to permit the implementation of PIMART will have far-reaching effects on the pharmacy profession. Another consideration to extend the pharmacist’s role would be to permit PCDT pharmacists to initiate and manage patients on ART and provide PrEP and PEP, after completion of the required training.

These examples illustrate how current policy both enables and constrains the ability of community pharmacists to provide HIV-related services in practice. Realising the full potential of DSD and pharmacy-based healthcare workers requires clear, enabling policies that support decentralisation, collaborative practice, and task-shifting, ensuring that all trained providers can practise to the full extent of their competencies to deliver timely, accessible, and person-centred HIV care.

## Conclusion

Currently, on-site nurses and pharmacists in community pharmacies are supported by remote medical practitioners providing services within their existing scopes of practice. Task shifting to NIMART- and PIMART-trained professionals positioned within community pharmacies could increase the rollout of ARVs, especially with regard to same-day initiation of HIV treatment and prevention therapies. This shift to alternate models of care will depend on the availability of healthcare professionals and resources (including laboratory services) as well as the cost of the service. In addition, nurses and pharmacists would need to understand and respect the limitations of their scopes of practice, working within their competencies and effectively using appropriate referral networks. There is, therefore, an urgent need for all healthcare professionals to advocate for patient-centred multi-disciplinary services instead of protecting dispensing and prescribing rights by profession.
